# Supplementary figures and images for: Protein Panel of Serum-Derived Small Extracellular Vesicles for the Screening and Diagnosis of Epithelial Ovarian Cancer
Source: Cancers (Basel). 2022 Jul 30;14(15):3719. doi: 10.3390/cancers14153719 (PMC9367436; doi:10.3390/cancers14153719)

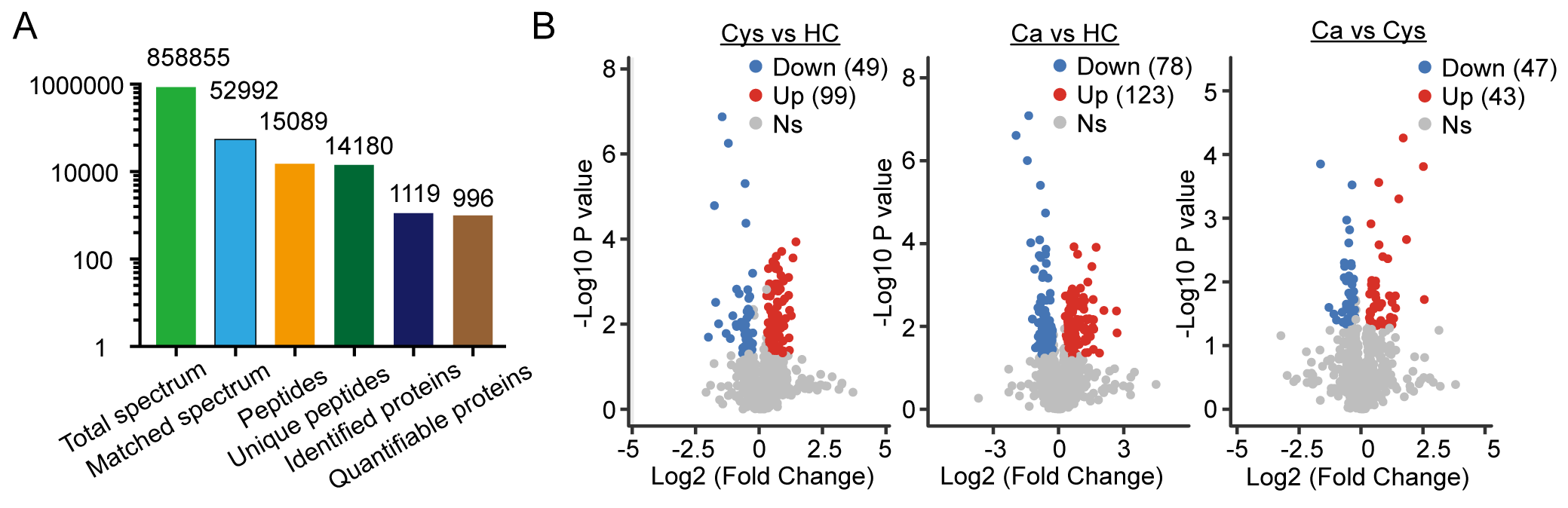

Supplement: Supplementary file 1 [file cancers-14-03719-s001.zip › Figure S1.tif]

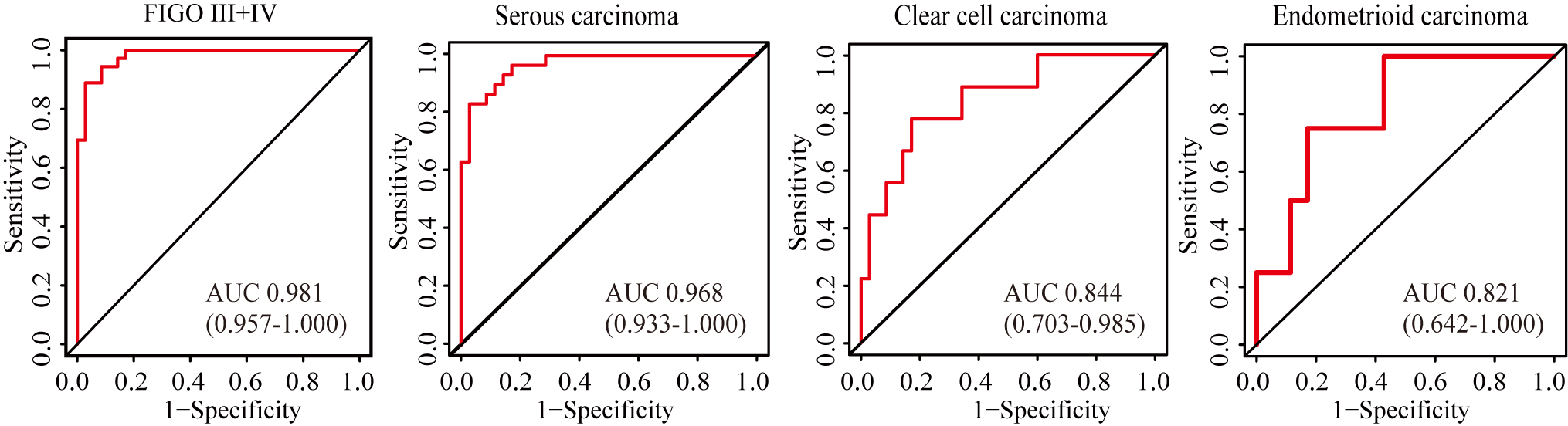

Supplement: Supplementary file 1 [file cancers-14-03719-s001.zip › Figure S2.tif]

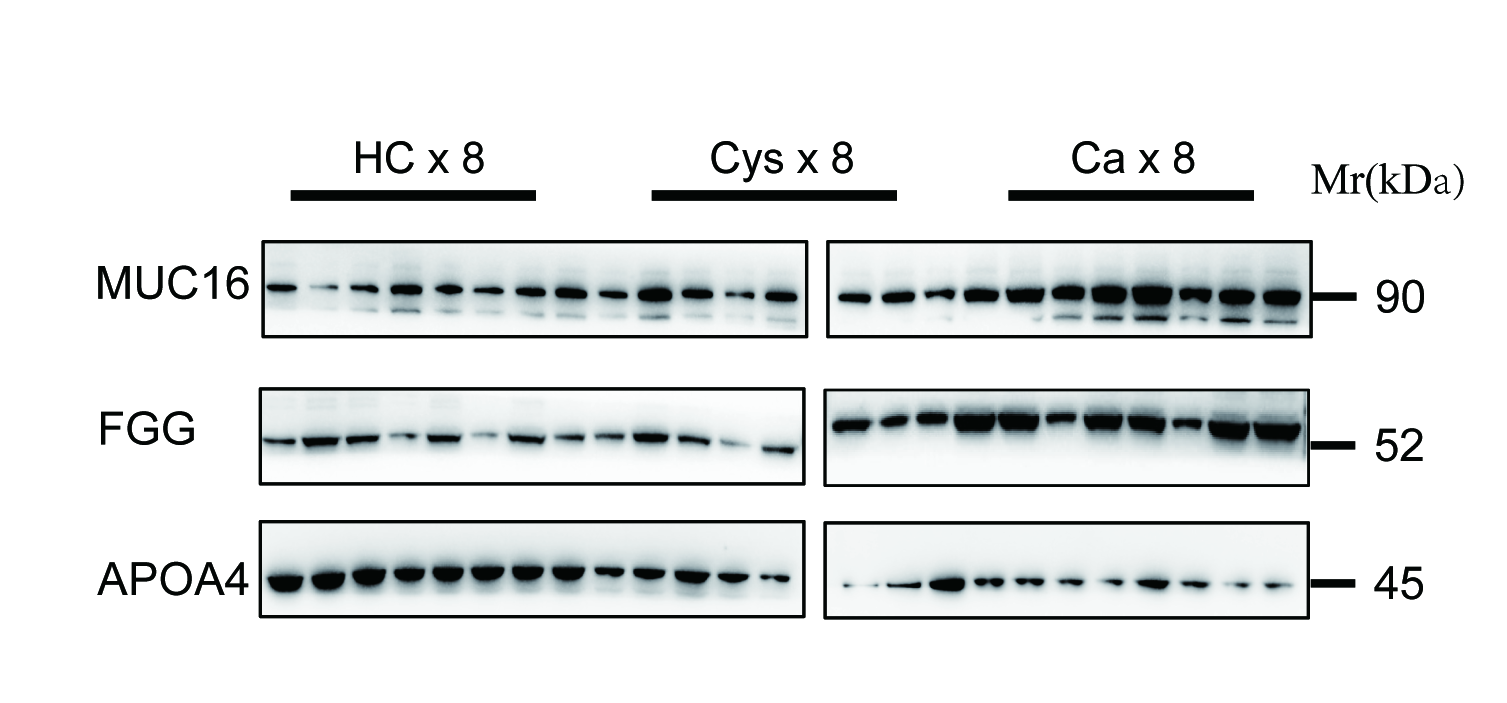

Supplement: Supplementary file 1 [file cancers-14-03719-s001.zip › Figure S3.tif]
